# Discriminating between Lysine Sumoylation and Lysine Acetylation Using mRMR Feature Selection and Analysis

**DOI:** 10.1371/journal.pone.0107464

**Published:** 2014-09-15

**Authors:** Ning Zhang, You Zhou, Tao Huang, Yu-Chao Zhang, Bi-Qing Li, Lei Chen, Yu-Dong Cai

**Affiliations:** 1 Department of Biomedical Engineering, Tianjin Key Lab of Biomedical Engineering Measurement, Tianjin University, Tianjin, P.R. China; 2 Institute of Health Sciences, Shanghai Institutes for Biological Sciences, Chinese Academy of Sciences and Shanghai Jiao Tong University School of Medicine, Shanghai, P. R. China; 3 Department of Genetics and Genomic Sciences, Icahn School of Medicine at Mount Sinai, New York, New York, United States of America; 4 Key Laboratory of Systems Biology, Shanghai Institutes for Biological Sciences, Chinese Academy of Sciences, Shanghai, P.R. China; 5 College of Information Engineering, Shanghai Maritime University, Shanghai, P.R. China; 6 Institute of Systems Biology, Shanghai University, Shanghai, P.R. China; Russian Academy of Sciences, Institute for Biological Instrumentation, Russian Federation

## Abstract

Post-translational modifications (PTMs) are crucial steps in protein synthesis and are important factors contributing to protein diversity. PTMs play important roles in the regulation of gene expression, protein stability and metabolism. Lysine residues in protein sequences have been found to be targeted for both types of PTMs: sumoylations and acetylations; however, each PTM has a different cellular role. As experimental approaches are often laborious and time consuming, it is challenging to distinguish the two types of PTMs on lysine residues using computational methods. In this study, we developed a method to discriminate between sumoylated lysine residues and acetylated residues. The method incorporated several features: PSSM conservation scores, amino acid factors, secondary structures, solvent accessibilities and disorder scores. By using the mRMR (Maximum Relevance Minimum Redundancy) method and the IFS (Incremental Feature Selection) method, an optimal feature set was selected from all of the incorporated features, with which the classifier achieved 92.14% *accuracy* with an *MCC* value of 0.7322. Analysis of the optimal feature set revealed some differences between acetylation and sumoylation. The results from our study also supported the previous finding that there exist different consensus motifs for the two types of PTMs. The results could suggest possible dominant factors governing the acetylation and sumoylation of lysine residues, shedding some light on the modification dynamics and molecular mechanisms of the two types of PTMs, and provide guidelines for experimental validations.

## Introduction

Post-translational modifications (PTMs) are crucial steps in protein synthesis and are important factors contributing to protein diversity. Among the various types of PTMs, lysine acetylation and sumoylation are emerging as two major types for both nuclear and cytoplasmic proteins, and they are related to several human diseases such as metabolic disorders and cancers [1]–[3].

Initially discovered on core histones approximately half a century ago, lysine acetylation has been found to be involved in multiple cellular processes such as transcriptional control, epigenetic program shaping, cytoskeleton organization, and energy metabolism regulation [4]–[7]. This type of reversible modification begins with the catalysis of lysine acetyltransferases (KATs, or histone acetyltransferases (HATs)), by adding the acetyl-group of an acetyl-CoA to the epsilon-amino group of an internal lysine residue. The process has been extensively characterized in many nuclear histones and transcription factors [8]. In contrast, lysine deacetylases (KDACs, or histone deacetylases (HDACs)) are responsible for the removal of acetyl groups [9]. Lysine acetylation and deacetylation have not only been associated with chromatin [4], [10] but have also been found to be related to cytoplasmic proteins in recent studies [5], [11].

Lysine sumoylation is another type of essentially reversible and highly regulated PTM. It occurs through covalent attachment of the small ubiquitin-like modifier (SUMO) to target proteins and is mediated by the activation of enzyme E1, conjugating enzyme E2, and ligase E3 [12]. Numerous chromatin-associated proteins have been found to be sumoylated [13]. Studies have revealed the impact of lysine sumoylation on transcriptional activation and repression, DNA replication and repair, and chromosome segregation, among other processes. Similar to methylation, the effects of lysine sumoylation are dichotomous, i.e. correlated with either gene activation or gene silencing [12], [14]. Additionally, sumoylation has been reported to act as a scaffold and facilitate the assembly of multiprotein complexes [15].

Because a lysine residue can undergo different PTMs, it is possible that there exists some cross-regulation among them [16]–[17]. For example, acetyltransferase p300 itself can block sumoylation of certain sites, which subsequently leads to the relief of transcriptional repression [18]. Nuclear receptor coregulators such as RIP140 also harbor various PTMs including acetylation and sumoylation, and their crosstalk may coordinate to direct RIP140 regulation [19]. To elucidate the cross-talk between acetylation and sumoylation, the first step is identifying the acetylation and sumoylation sites in proteins.

However, traditional experimental methods including mass spectrometry and Chip-on-Chip [20] techniques are often time consuming, expensive, and sometimes insufficient to recognize all of the modification sites in proteins. Computational methods could complement experimental methods by predicting potential target sites, revealing consensus motifs and providing insight into the molecular mechanisms of the modifications. Several methods for the prediction of lysine acetylation sites [9], [21]–[24] and sumoylation sites [25] have been developed. However, most of these prediction approaches have focused on predicting only one type of PTM site, i.e., either acetylation or sumoylation sites, thus providing little information about their crosstalk. In this study, we developed a computational method to discriminate between sumoylation sites and acetylation sites. We also performed an analysis of the optimal features selected in the method, which could suggest possible differences between the two types of PTMs, shedding some light on the governing factors and their molecular mechanisms.

## Methods

**Figure 1:**
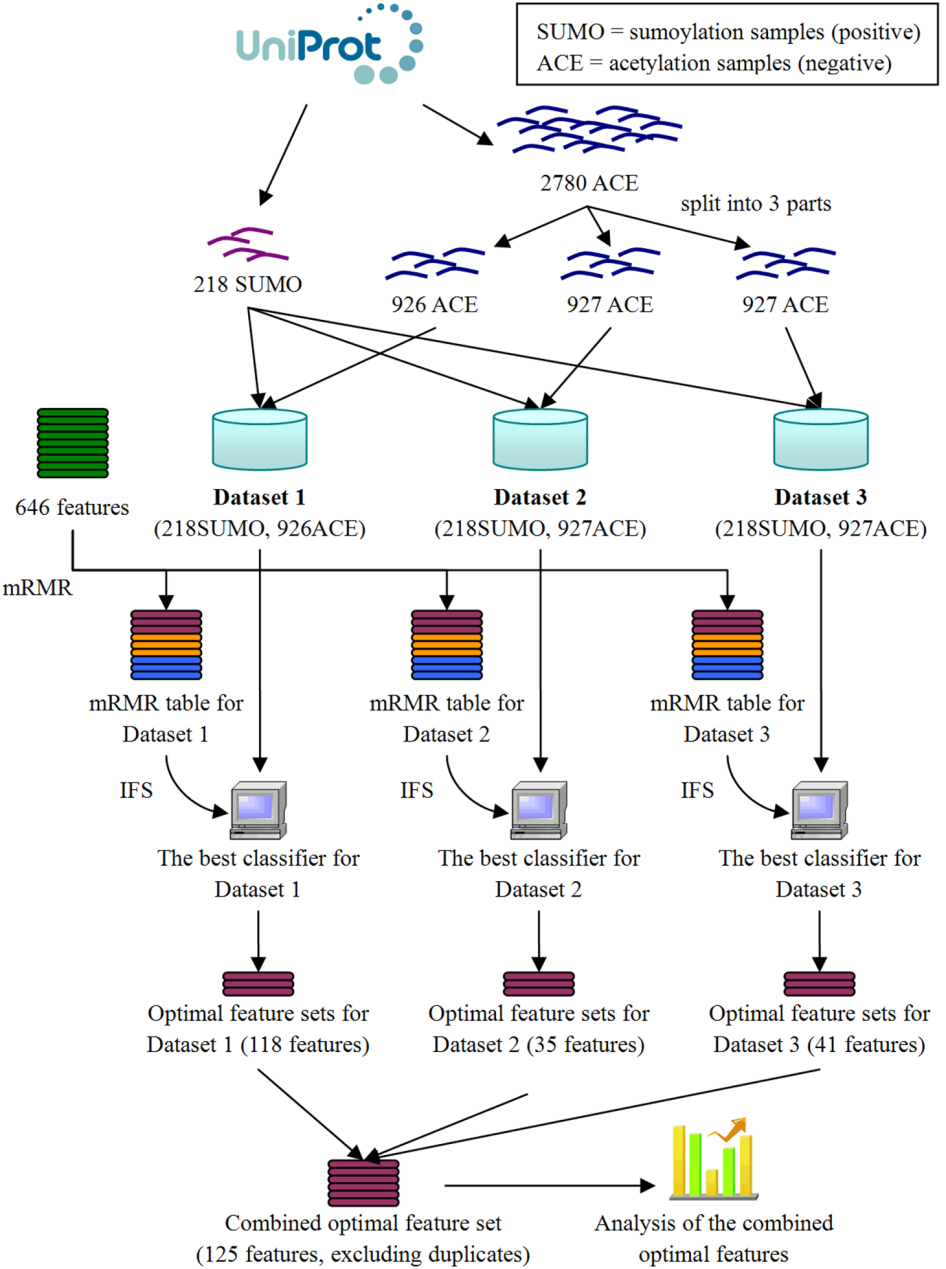
Flowchart representing the entire workflow of this study. All samples were downloaded from UniProt. The acetylation samples were separated into 3 parts. Then, every one of the 3 parts was combined in turn with all of the sumoylation samples to generate a dataset. In total, 3 datasets were generated. In each dataset, an optimal feature set was selected based on the mRMR and the IFS approach. The 3 optimal feature sets were combined and analyzed.

The entire workflow of this study is illustrated in **Fig. 1**.

### Dataset

All of the acetylation and sumoylation proteins used in this study were obtained from the UniProt database (http://www.uniprot.org/, release 2013_07). Proteins without experimentally verified modification residues and with sequence identities >40% were removed. We also removed sequences with lengths >2700 (e.g., P78527, Q96PK2, and Q9Y520) because SSpro4 software [26], which was used in this study to calculate protein secondary structures, cannot be run on proteins with lengths >2700. However, secondary structure was one type of feature that was necessarily used to construct our model. A small set of proteins, whose sequences contained non-standard residues not belonging to the 20 common amino acids such as ‘X’ (e.g., P83865), were also removed. Finally, 1677 proteins remained, among which there were 2780 acetylation sites (1566 proteins had acetylation sites) and 218 sumoylation sites (138 proteins had sumoylation sites); 27 proteins had both acetylation sites and sumoylation sites. There was no site that was both an acetylation site and a sumoylation site. The dataset is given in **File S1**.

Similar to development of PTM site predictors [27]–[32], in the present study, the sliding window strategy was utilized to extract positive and negative peptide samples. In our previous work, we predicted sumoylation sites and achieved 89.18% accuracy by only extracting 6 residues upstream and 6 residues downstream of the sumoylation sites [31]. We also provided a biological analysis of sumoylation, which suggested that the most important sites in determining whether a peptide would be sumoylated were the 7th, 4th, 1st, 2nd, and 3rd sites [32]. Shi et al. [33] used −6∼+6 region surrounding the center lysine to develop PLMLA to predict acetylated lysine residues. Gnad et al. [24] used 2 to 8 amino acids upstream and downstream of the center lysine to predict acetylation sites. It has been shown in structural studies that peptide substrates coupled with lysine acetyltransferase (KAT) domains do not exceed 14–20 amino acids in length [17], [34]. In summary, to the best of our knowledge, both sumoylation and acetylation motifs should have a length of no more than 21. Therefore, we adopted a window length 21 in this study to investigate both types of PTMs; this window length was also successfully used in our previous studies to predict several other types of PTMs [27]–[30].

By sliding a 21-residue window along each protein sequence, we extracted 21-residue peptide segments centered on a sumo-lysine or on an acetyl-lysine residue, with 10 residues upstream and 10 residues downstream of the center lysine. Peptide segments with lengths less than 21 were complemented by adding blank sites whose features were set to 0. In this study, peptides with a centered sumo-lysine were regarded as positive samples, while peptides with a centered acetyl-lysine were regarded as negative. Accordingly, 218 positive and 2780 negative samples were extracted.

The dataset was unbalanced due to an extremely high number acetylation samples. Therefore, we randomly split the set of 2780 acetylation samples into three parts without overlaps. There were 926, 927, 927 acetylation samples in the three parts. The 218 sumoylation samples were combined with the 3 parts of acetylation samples to generate 3 datasets, respectively. In each dataset, all 218 sumoylation samples were presented with one of the 3 parts of acetylation samples. The 3 datasets were named as Dataset 1, Dataset 2, Dataset 3.

### Feature extraction

We used the following features to encode all of the 21-residue peptides, for both the positive and negative samples.

#### Features of PSSM conservation scores

It is widely believed that the evolutionary conservation observed in multiple sequence alignments is important in biological sequence analysis [27]. A conserved residue could be under strong selective pressure and thus could play a vital role in protein function. In this study, the conservation status of a residue in a peptide was measured using Position Specific Iterative BLAST (PSI-BLAST) [35], which is a powerful sequence searching method. This method was used to search the UniRef100 database (Release: 15.10, 03-Nov-2009) through 3 iterations with 0.0001 as the E-value cutoff. For each residue in a peptide, a 20-dimensional vector was computed to denote the probability of the residue against its mutations for the 20 types of native amino acids. Therefore, for a 21-residue peptide, all such 20-dimensional vectors for the 21 residues in the peptide composed a matrix, called position specific scoring matrix (PSSM), which can be used to quantify the conservation status of every residue in a peptide. These 20×21 = 420 values in the matrix (called PSSM conservation scores) were used in this study as one type of feature to encode a peptide to construct our classifier.

#### Features of amino acid factors

The 20 native amino acids have different physicochemical and biochemical properties. Different compositions of the 20 native amino acids in a protein may endow the protein with different physicochemical and biochemical properties and thus affect protein structure and function. The AAIndex [36] is a database containing the physicochemical and physiological properties of the 20 amino acids. Atchley et al. [37] performed multivariate statistical analyses on the database and generated 5 different numerical patterns for each amino acid to reflect their five properties: codon diversity, electrostatic charge, molecular volume, polarity and secondary structure. Herein, we used the 5 numerical scores for each residue in a 21-mer peptide, called amino acid factor features, as another type of feature to construct our model.

Note that because the center residue in a 21-mer peptide was always lysine, it was not necessary to incorporate the numerical scores of the centered lysine. Only the 20 surrounding residues should be encoded. Therefore, there were only 5*20 = 100 amino acid factor features for one 21-mer peptide.

#### Features of secondary structures

Protein secondary structures are of great importance in residue modifications [17] and should also be employed to construct classifiers. In this study, the secondary structure state of every residue in a 21-mer peptide was calculated using SSpro4 [26]. SSpro4 can predict the secondary structural state of every residue in a protein and give 3 different ‘helix’, ‘strand’, or ‘other’ states for every residue. To transform the 3 different secondary structure states to numeric features, we represented each of the states as a 3-bit binary value. The ‘helix’, ‘strand’ and ‘other’ states were denoted as ‘100’, ‘010’ and ‘001’, respectively. A 3-bit binary value can be regarded as comprising 3 numeric features. For example, ‘100’ can be regarded as the 3 numeric features 1, 0 and 0. Therefore, there were 3×21 = 63 secondary structure features for a 21-length peptide, although each of these 63 features was either 0 or 1. These 63 features for a peptide were also used as another type of feature to construct our classification model.

#### Features of solvent accessibilities

We also took into account residue solvent accessibility, because the effects of solvent accessibilities on residue modifications have been demonstrated by previous studies [38]. We used SSpro4 [26] to compute the solvent accessibilities of every residue in a 21-residue peptide. SSpro4 can give a ‘buried’ or ‘exposed’ categorization for every residue. To transform the 2 different solvent accessibility states to numeric features, we represented each of the 2 states as a 2-bit binary value. The ‘buried’ and ‘exposed’ states were denoted as ‘10’ and ‘01’, respectively. A 2-bit binary value can be regarded as 2 numeric features. Therefore, there were 2×21 = 42 solvent accessibility features for a 21-length peptide, although every one of these 42 features was either 0 or 1. These 42 features for a peptide were also used as another type of feature set to construct our model.

#### Feature of disorder scores

If a region of a protein is devoid of stable structure, or if it has a large number of conformations, it is called a “disordered region”. Disordered regions could play important roles in protein structure and function [28], [39]–[40]. Disordered regions always contain more PTM sites than non-disordered ones; therefore, the disordered states of a protein are quite important in PTM studies. The likelihood of one residue forming a disordered structure can be measured by VSL2 software [41]. VSL2, one of the best disorder predictors [41], can give a disorder score for every residue in a peptide. The higher the score is, the more likely that the residue forms a disordered structure. We computed the disorder score for every residue in a 21-residue peptide and used the 21 scores as another type of feature set to construct our model. There were only 21 features of disorder scores for a peptide because each residue only had one score value.

To summarize, the features utilized in this study are listed in **Table 1**. As seen in **Table 1**, for a 21-length peptide, there are 420 PSSM conservation score features, 100 amino acid factor features, 63 secondary structure features, 42 solvent accessibility features and 21 disorder score features. A total of 646 features were extracted for such a 21-length peptide. This method was quite similar to that used in [28] for predicting protein γ-carboxylation sites, as well as to that used in [27] for predicting protein pyruvoyl-serine sites.

**Table 1:** Features utilized to encode a 21-residue peptide.

Feature type	Features	Number
PSSM conservation scores	20-dimensional vector	420
Amino acid factors	Polarity, secondary structure, molecular volume, codon diversity, electrostatic charge (only for surrounding sites, except the center)	100
Secondary structures	Secondary structures: helix, strand, other	63
Solvent accessibilities	Solvent accessibilities: buried, exposed	42
Disorder scores	Disorder score reflecting the disorder status of the residue	21
Total		646

### Feature selection

We employed the mRMR (Maximum Relevance Minimum Redundancy) method [42]–[44] to rank the importance of the 646 features, according to the Maximum Relevance Minimum Redundancy criterion. The Maximum Relevance criterion selects features most related to the target. The Minimum Redundancy criterion excludes features containing redundant information among the selected features. Briefly, to rank features using mRMR criteria, two values were calculated for each feature: value A for relevance and value B for redundancy. Then, the value A–B is used to measure the feature; the higher the value A–B is, the higher the feature ranks. For details of the mRMR method, please refer to [27]–[28], [42]–[44].

Using this method, the 646 features were ordered. In the ordered list, called the mRMR table, a feature with a smaller index indicated that it had a better trade-off between the maximum relevance and the minimum redundancy and thus could be more important. Based on the ordered feature list, a series of classifiers can be constructed by using different features. For example, a classifier can be constructed by using only the top 1 feature from the list. By using the top 2 features from the list, another classifier can be constructed, and so on. The classifier of the next round always contained 1 more feature from the ranked list, following the previous round. In this procedure, features in the ranked feature list were added one by one in decreasing order of rank. A new feature set was generated when another feature had been added, and for each of the feature sets, a classifier was constructed. If there were 646 features in the list, a total of 646 classifiers could be constructed. This procedure is called the IFS (Incremental Feature Selection) method [45]–[46]. The 646 classifiers constructed use the first feature, the first 2 features, the first 3 features, and so on, up to all 646 features, respectively, from the ranked feature list. From the 646 classifiers, we can select the best one to discriminate the two modifications, based on which had the best performance, and the features used by that classifier were regarded as composing the optimal feature set.

### Prediction methods

We employed the Random Forest (RF) algorithm to construct the classifier. Developed by Loe Breiman [35], RF is a popular machine-learning algorithm that has recently been successfully applied in various biological problems [27]–[28], [47]. As an ensemble classifier, the RF method contains several decision trees. The final classification result is determined by the class with the most votes among all of the trees. For a detailed description of the RF algorithm, please refer to [48]–[49]. In this study, the Random Forest classifier in Weka 3.6.4 [50] software was employed to perform the prediction. The algorithm was run with default parameters.

### Performance measurements

In this study, we used the jackknife cross-validation test to assess the efficiency of our classifier, witch was regarded as the most objective among various evaluation methods.

The following measurements were used in this study:



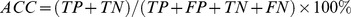
(1)

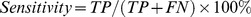
(2)

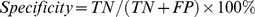
(3)

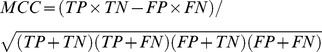
(4)


in which *TP*, *TN*, *FP*, *FN* denoted the number of true positives, true negatives, false positives and false negatives, respectively.

When measuring the performance of a classifier, it is important to note that a naive method could make use of the composition of the data to label all instances as the dominant class (acetylation in this study), resulting in an *accuracy* (*ACC*) equal to the percentage of instances of that class (e.g., 926/(218+926) = 80.94% in our Dataset 1). In actuality, such a naive method is useless, although its *accuracy* may be high. Therefore, several other measurements must be used in addition to the *accuracy*.

*Sensitivity* is the percentage of positive samples (sumoylation) that are correctly classified as positive by the method. By contrast, *specificity* is the percentage of negative samples (acetylation) that were correctly classified to be negative. *Sensitivity* and *specificity* values <100% reflect the occurrence of false-negative and false-positive errors of the method, respectively.

*MCC* (Matthews Correlation Coefficient), first used in Matthews’s study [51], is a single-valued but robust measurement of performance. The *MCC* value ranges from −1.0 to +1.0, where 0 represents a random correlation between the classified variables and the actual variables, +1.0 a perfect correlation, and −1 a perfect negative correlation [52]–[54]. *MCC* takes into account both false-positive and false-negative errors and is generally deemed to be a balanced measurement even if the classes are of very different sizes [17]. For these reasons *MCC* is more reliable than *accuracy*. Therefore, the *MCC* was used throughout this study as the main evaluator and has also been used to assess PTM prediction methods in the literature, such as in [17], [27]–[28].

## Results

### Feature selection and the optimal classification

We employed the mRMR (Maximum Relevance Minimum Redundancy) method [42]–[44] to rank the importance of the 646 features. Using this method, the 646 features were ordered in the generated mRMR table. Because there were 3 datasets in this study, 3 iterations were performed with each of the 3 datasets. Therefore, 3 mRMR tables were obtained, one for each dataset. The 3 mRMR tables are provided in **File S2**.

**Figure 2:**
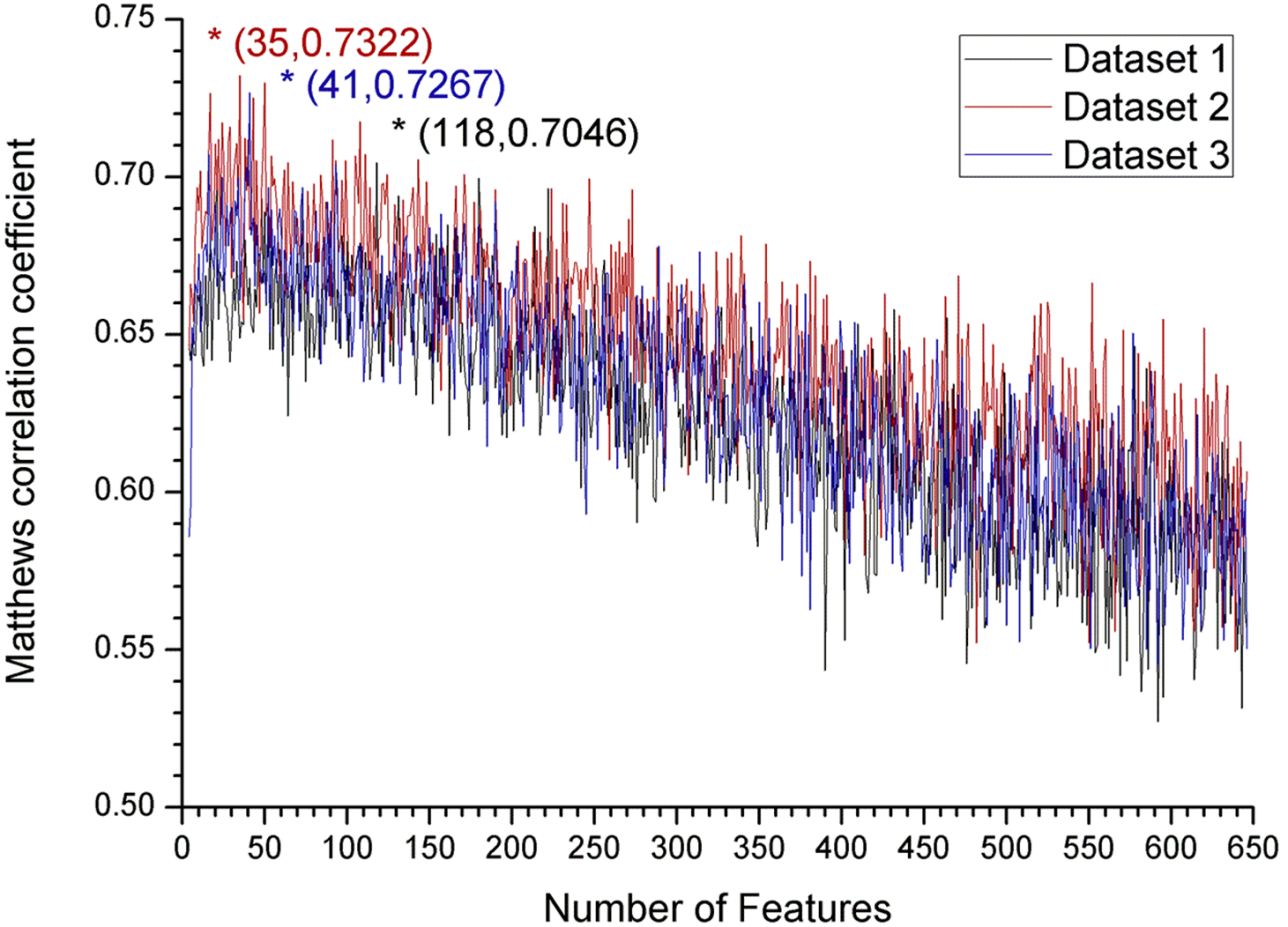
The IFS curves for the 3 datasets. A series of classifiers can be constructed using different number of top features from the mRMR tables during the IFS process. Plot showing the performances of the different classifiers, with *MCC* as the main measurement on the y-axis. As the classifiers used different numbers of features, we represented the classifiers with the corresponding number of features used in x-axis. In Dataset 1, the highest *MCC* (0.7046) was achieved at 118 features. This finding demonstrated that the classifier adopting the top 118 features in the mRMR table for Dataset 1 performed the best, and the 118 features were regarded as composing the optimal feature set for Dataset 1. Similarly, a peak of *MCC* 0.7322 and 0.7267 was obtained at 35 and 41 features in Dataset 2 and Dataset 3, respectively. These results demonstrated that by using the top 35 and 41 features in the mRMR table for Dataset 2 and Dataset 3, respectively, the classifier performed the best. The 35 and 41 features were regarded as composing the optimal feature set for Dataset 2 and Dataset 3, respectively.

In the IFS (Incremental Feature Selection) procedure, 646 classifiers were constructed using the first 1 feature, the first 2 features, the first 3 features, and so on until all 646 features were used, respectively, from the ranked feature list in the mRMR table. Then, the performance of each of the 646 classifiers was measured according to *ACC*, *sensitivity*, *specificity* and *MCC*. The performance results of the classifiers can be found in **File S3**. We used *MCC* as the main evaluator to measure the performances of the classifiers. We plotted the *MCC*s against different classifiers in **Fig. 2** to show the performances of the classifiers; the resulting curve is called the IFS curve. As the classifiers used different number of features, we represented the classifiers on the x-axis with the corresponding number of features they used.

The optimal feature set, with which the corresponding classifier yielded the best performance, can be obtained. From **Fig. 2** and the data in **File S3**, it can be observed that in Dataset 1, the classifier adopting the top 118 features performed the best, with an *MCC* of 0.7046. In Dataset 2, the best classifier was the one using the top 35 features, yielding an *MCC* of 0.7322. In Dataset 3, the *MCC* reached a maximum of 0.7267 when the classifier was constructed using the top 41 features. The *MCC* values and the *SN*, *SP*, *ACC* measurements for the best classifiers in the 3 datasets, respectively, are summarized in **Table 2**. The 118, 35 and 41 features were regarded as composing the 3 optimal feature sets for the 3 datasets, respectively. The detailed features of the 3 optimal feature sets can be found in the mRMR table in **File S2**.

**Table 2:** The classification performances of the 3 best classifiers for the 3 datasets.

	Optimal Features	*SN*	*SP*	*ACC*	*MCC*
Dataset 1	118	66.51%	97.30%	91.43%	0.7046
Dataset 2	35	71.10%	97.09%	92.14%	0.7322
Dataset 3	41	68.35%	97.63%	92.05%	0.7267

From **Table 2**, it is clear that the successful classification indicated that the optimal features are capable of distinguishing the two types of PTMs: lysine sumoylation and lysine acetylation. The features selected in the optimal feature sets reflect the differences and governing factors of the two types of PTMs. Analysis of the features may shed some light on the mechanisms of their formations.

### The combined optimal feature set

We combined the 3 optimal feature sets for the 3 datasets, excluding duplicates features. Finally, 125 optimal features were obtained, which can be found in **File S4**. These 125 optimal features were analyzed and are discussed below, because features that can be optimally used to discriminate acetylation and sumoylation are good candidates for analyzing the differences between them.

**Figure 3:**
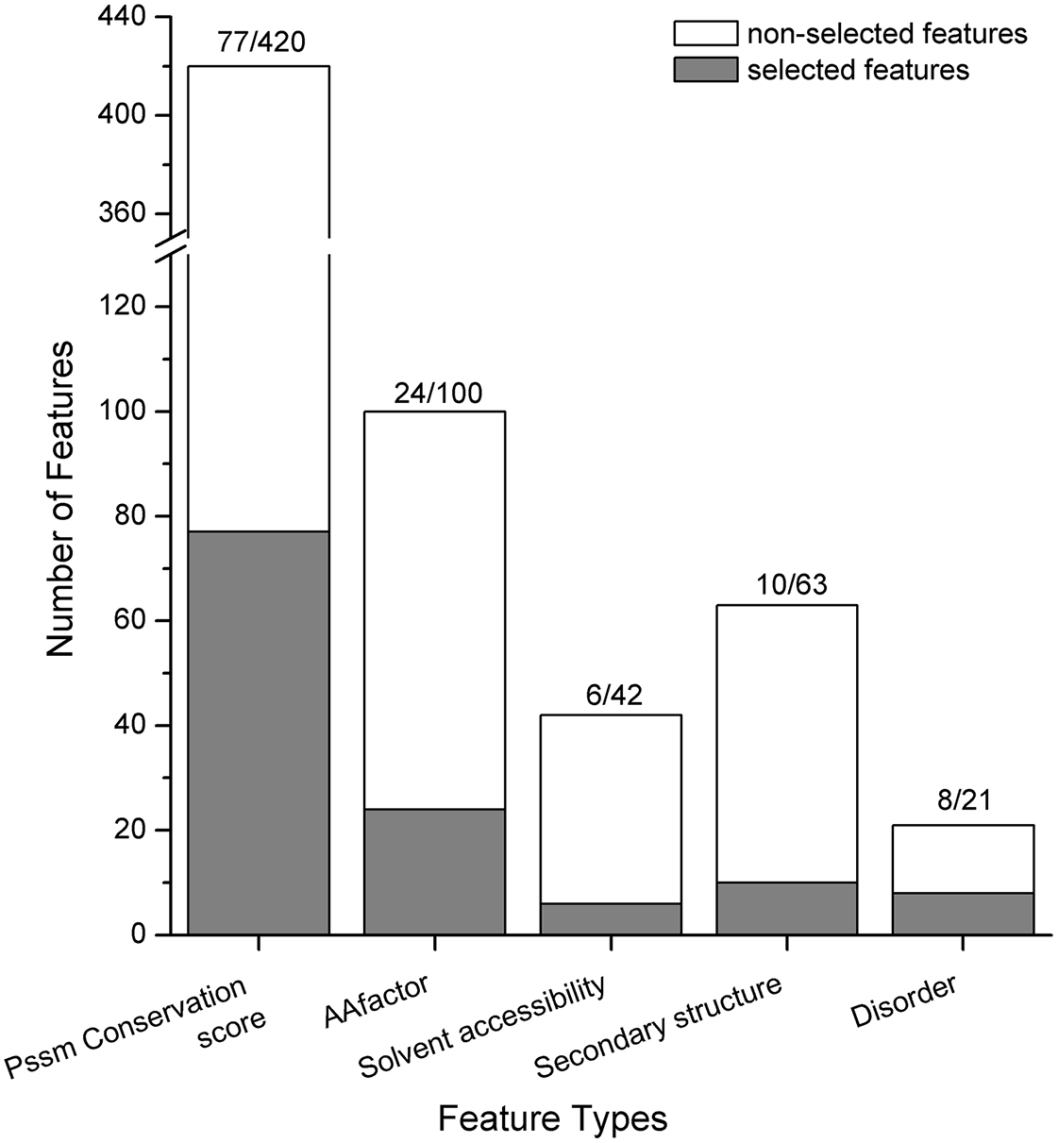
Type distributions of the 125 combined optimal features. The 125 features were obtained by combining the 3 optimal feature sets for the 3 datasets. The histograms show the number of optimal features belonging to each type, as well as the total number of features of each type.

We examined the feature type of the combined 125 optimal features, and the feature type distributions are depicted in **Fig. 3**. It can be observed in **Fig. 3** that of the 125 optimal features, 77 belonged to the PSSM conservation score, followed by 24 belonging to the amino acid factor, 6 belonging to the solvent accessibility, 10 belonging to the secondary structure and 8 belonging to the disorder. PSSM occupied the majority of the optimal features (61.6%), and amino acid factor was the second highest (19.2%), indicating their prominent roles in discriminating acetylation and sumoylation modifications.

## Discussion

### Optimal feature type analysis

#### PSSM features

**Figure 4:**
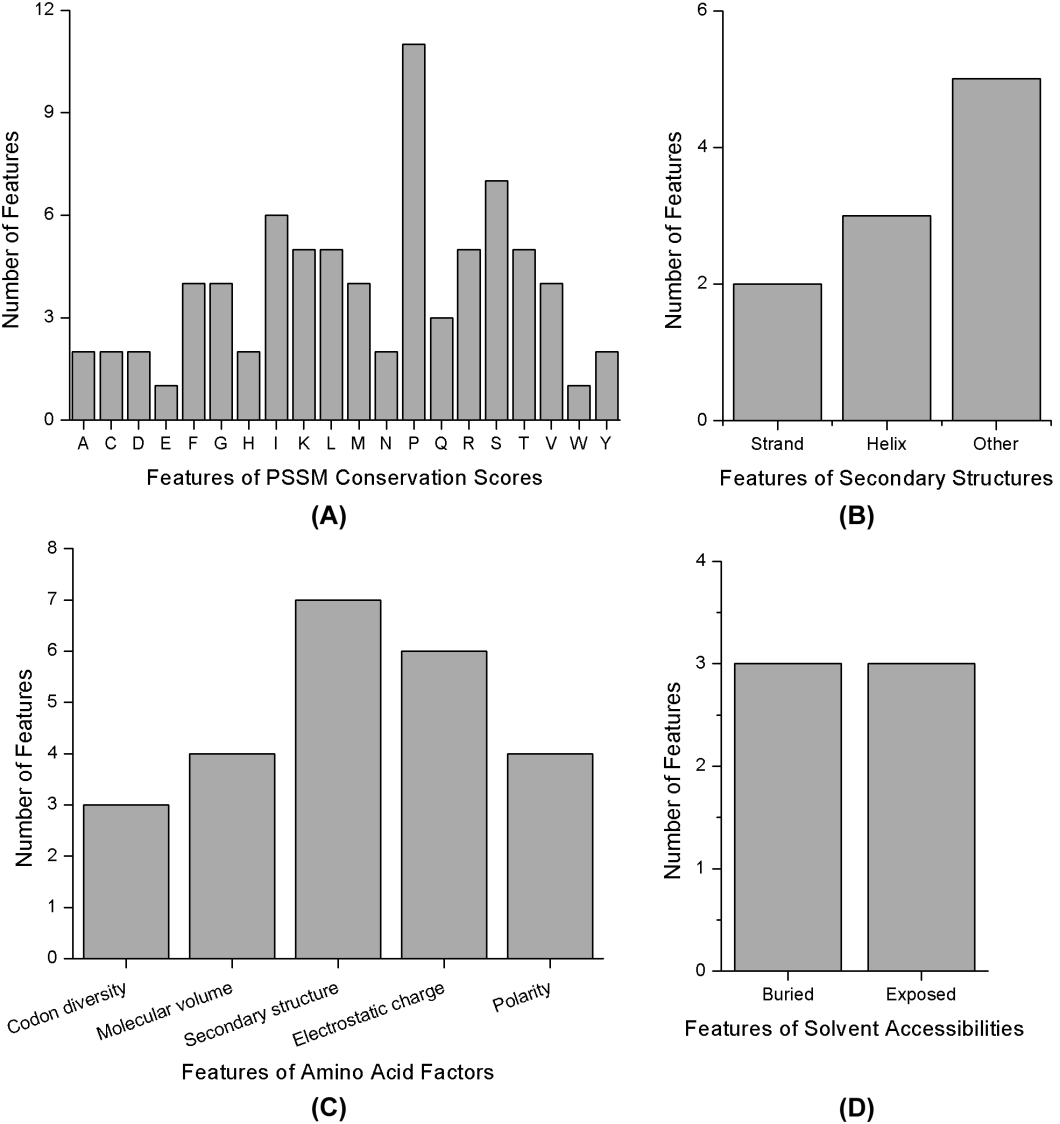
Sub-type distributions of the 125 combined optimal features. Histogram showing (A) the distribution of subtypes of the conservation score features; (B) the distribution of subtypes of the secondary structure features; (C) the distribution of subtypes of the amino acid factor features; (D) the distribution of subtypes of the solvent accessibility features.

The distribution of the selected PSSM features against mutations of 20 native amino acids is shown in **Fig. 4(A)**. The mutations of 20 different amino acids could have different impacts on discrimination between acetylation and sumoylation. Mutations to P (Proline), S (Serine) and I (Isoleucine) could affect the most, with more than 6 features. As proline is the residue most commonly found near interaction sites, protecting the integrity of the sites [55], it is suggested that the two modifications would show a distinct difference in the conservation of proline. Proline is frequently involved in acetylation and plays an important role [56]–[57]. In contrast, while a mutation of proline-90 in small ubiquitin-related modifier (SUMO) genes is fatal for both hydrolase and isopeptidase activities of SUMO peptidases in humans [58], there is limited evidence supporting a link between prolines and sumolyzation. However, some SUMO targets, such as estrogen receptor β, are subjected to SUMO modification, depending on phosphorylation of its serine residues [59].

#### Amino acid features

Sub-type distributions of amino acid factor features in the 125 optimal features are depicted in **Fig. 4(C)**. It was found that secondary structure and electrostatic charge were the most important, while molecular volume, polarity and codon diversity contributed almost equally. In principle, the protein-protein interaction interface is determined by the amino acid types and the secondary structures of residues in the interface [60]. Secondary structures have been accepted to be essential for protein-protein interactions and appear to be useful for the characterization and classification of the interacting sites [61]. As acetylation and sumoylation are both enzymatic and reversible, the importance of secondary structure here might suggest again the different interaction modules of enzymes and targets between them. Additionally, the secondary structure feature of site 13 always ranked at first in the 3 mRMR feature lists, suggesting that the secondary structure of this site could be quite different between acetylation and sumoylation. Furthermore, the electrostatic charge feature of site 10 ranked above 12 in all the 3 mRMR feature lists, indicating that it could show much difference between acetylation and sumoylation.

#### Other features

Though only a few features were selected in the PSSM conservation score and amino acid factor in quantity, secondary structure features still made up the subordinate portion. It can be seen from **Fig. 4(B)** that “other” non-regular structures were more important than “strand” and “helix” regular structures, perhaps because the flexibility of non-regular structures allow the protein with an easy fit into enzyme catalytic sites [62]–[63]. Because previous studies have shown that some types of post-translational modifications prefer to occur in coiled regions [6], [11], [17], [64]–[65], our result supported the aforementioned finding that these two modifications occupied different modes of action. A much higher ratio of disorder features (8 out of 21) was selected than other feature types (**Fig. 2**), suggesting the importance of disorder in the topology of protein modifications as well as in protein-protein interactions [62].

In contrast, there were only 6 solvent accessibility features selected in the optimal feature set, accounting for a small fraction; there was no difference between buried and exposed solvent accessibility features, as observed in **Fig. 4(D)**. Several reports have demonstrated that both acetylation and sumoylation are prone to utilize hydrophobic residues as dominant residues for their modifications [63], [66]–[69]. It is suggested that solvent accessibility may not be a very efficient feature for distinguishing the two types of modifications.

### Optimal feature site analysis

**Figure 5:**
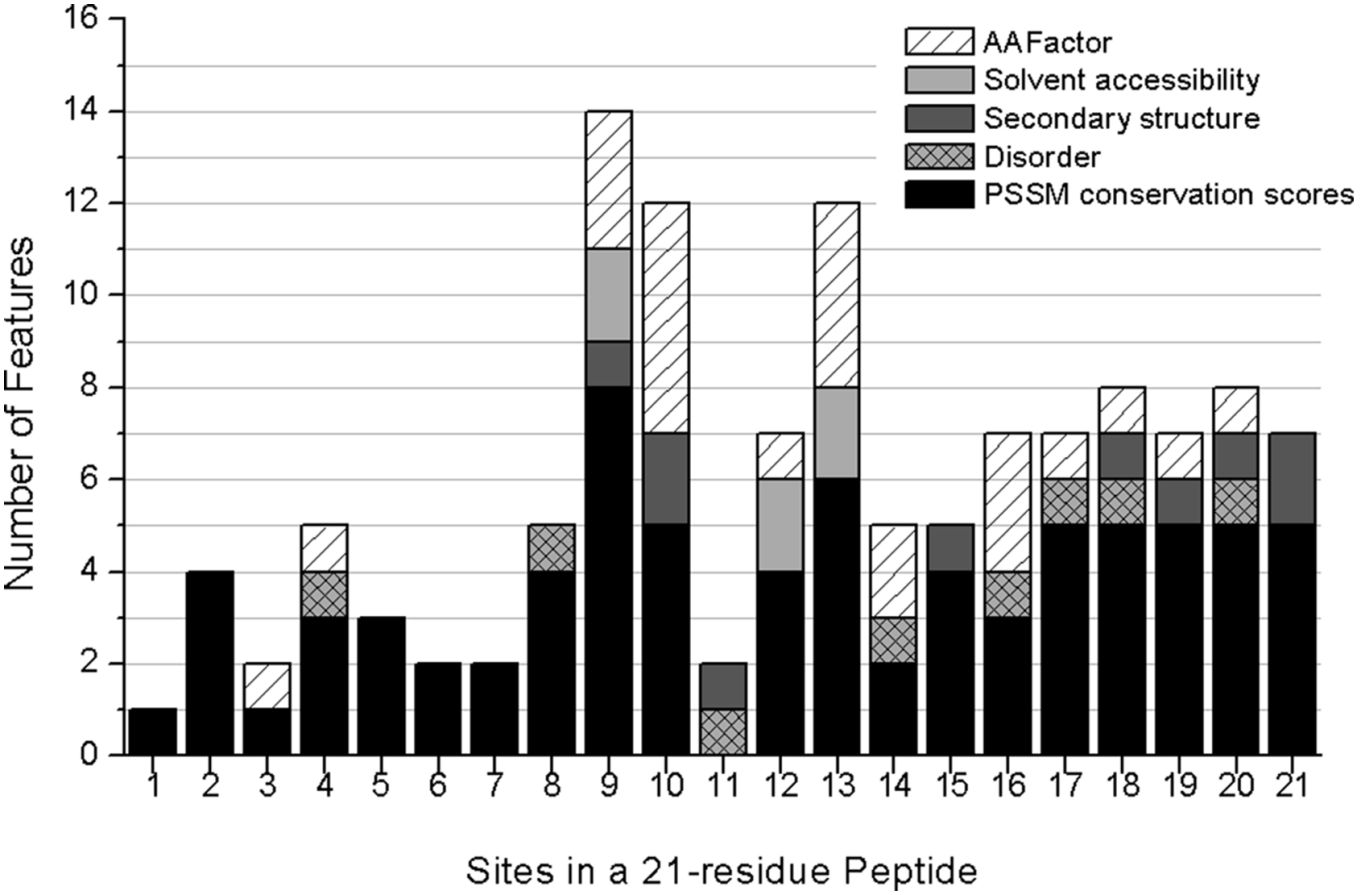
Site distributions of the 125 combined optimal features. The solid bars, checked bars, gray bars, light gray bars and hatched bars represent features of PSSM conservation scores, disorder, secondary structures, solvent accessibility and amino acid factors, respectively.

To investigate whether there was a certain pattern around the modified lysine site to determine acetylation or sumoylation, we analyzed the site distribution of the 125 optimal features, and the results are depicted in **Fig. 5**.

It can be observed that sites 9, 10 and 13 accounted for the most features, suggesting their important roles in discriminating the two modifications. The selected optimal conservation features also largely resided at sites 9 and 13, suggesting the conservation differences in the protein sequences at these two sites. From **Fig. 5**, it also can be observed that amino acid factor features at site 10 contributed the most, followed by sites 9, 13, 14 and 16. However, features at site 1 contributed the least (only 1 optimal feature).

It is also worth pointing out that none of the “hot” sites, including 9, 10 and 13, contained disorder features, while site 11 only had secondary structure and disorder features. One possible explanation might be that the disorder status at specific sites plays a vital role in determination of different modifications required for forming specific conformations and bind specific enzymes, which again reinforces the importance of including protein structures in post-translational modifications. From **Fig. 5**, it was also demonstrated that irrespective of feature types, features downstream of the center lysine were much more important than those upstream, especially for the disorder and amino acid factor features. This finding suggested that we should pay more attention to the downstream sequence of the center modified lysine in cross-talk studies of acetylation and sumoylation in future.

### Occurrence frequencies of amino acids

**Figure 6:**
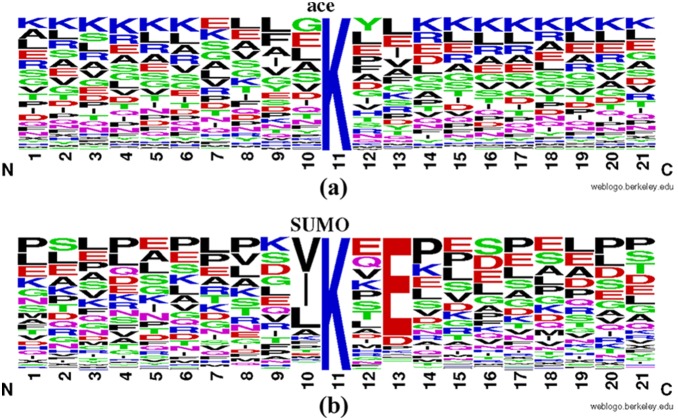
Amino acid occurrence frequencies surrounding the active-lysine generated by WebLogo [70]. Logo illustrations were generated based on all of the 21-residue peptides in our dataset, showing the occurrence frequencies of 20 amino acids surrounding the sumo-lysine (a) or the acetyl-lysine (b). N and C represented the N- and C-terminuses of the 21-residue peptides, respectively.

Occurrence frequencies of 20 native amino acids surrounding the acetylation and sumoylation sites were each represented with WebLogo [70] (http://weblogo.berkeley.edu/) (and shown in **Fig. 6**). It can be observed that consistent with previous reports, the preferred motif of acetylation emphasized the great importance of amino acid K [71]. However, only a few amino acid preferences for sumoylation can be found in its consensus motif ΨKXE (where Ψ represents an aliphatic amino acid, and X is any amino acid) [25], [72]. These findings also corroborated the finding that site 13 (domination of E in sumoylation) had a large number of features belonging to the PSSM mentioned above (**Fig. 5**). Combined with our mRMR result, in which the secondary structure feature of site 13 ranked the first in the optimal feature set (see **File S2**), it could be inferred that site 13 was a strong governing factor to the discrimination of the two modifications. Interestingly, site 10 also contributed, with a high frequency of G and E for acetylation, but V and I for sumoylation.

### Guidelines for experimental validation

Until now, few reports available have distinguished acetylation and sumoylation of proteins *in* *silico*; therefore, it is worth noting that the selected optimal features at different sites from this study could provide useful clues for experiments to identify the differences and cross-talks between the two modifications. Among all of these optimal features, the PSSM conservation scores were determined to be the most important, followed by the amino acid factors (**Fig. 3**). It was also suggested that secondary structures and electrostatic charges of amino acids at sites 9, 10, 13 and 16 played pivotal roles (**Fig. 5**). Although both acetylation and sumoylation usually occurred within coiled regions, they could be a part of different interaction modules, and therefore, more non-regular secondary structure features should be taken into consideration (**Fig. 4(B)**). In addition, many studies have found that both Acetyl- and SUMO-interacting motifs are hydrophobic; however, the solvent accessibility features (buried or exposed) were not efficient enough to discriminate (**Fig. 3(D)**). Moreover, acetylation and sumoylation showed differential preferences in terms of amino acid frequency. K was prone to appear in the acetylation flanking sequences, while only a few amino acids showed strong conservation, such as E at site 13 in sumoylation (**Fig. 6**). Accordingly, these optimal features could be good candidates for validation by experiments and further investigations.

## Conclusion

In this study, we analyzed the factors discriminating sumoylation and acetylation by constructing classifiers and using hybrid features of sequences: PSSM, amino acid factors, secondary structures, solvent accessibilities, and disorder scores. Our results were consistent with consensus motifs previously found for acetylation and sumoylation. The results of the feature analysis from this work might contribute to an understanding of the mechanisms of lysine acetylation and sumoylation and provide guidance for related experiments for validations.

## Supporting Information

File S1 The dataset used in this study.
(ZIP)

File S2 The mRMR feature tables for the 3 datasets. The top 118, 35 and 41 features in the 3 tables are composed of the 3 optimal feature sets for the 3 datasets, respectively.
(XLS)

File S3 The IFS results for the three datasets. Note that the data are not shown for classifiers with feature numbers below 3 because these classifiers yielded no results for feature sets that were too small.
(XLS)

File S4 The 125 combined optimal features from 3 optimal feature sets for 3 datasets.
(XLS)
